# Nursing students’ mindfulness and emotional regulation: an integrative review

**DOI:** 10.1590/0034-7167-2023-0466

**Published:** 2024-09-06

**Authors:** Daiana Alves Vendramel da Costa, Cássia Janne Nonato da Costa, Moisés Kogien, Larissa Bessani Hidalgo Gimenez, Edilaine Cristina da Silva Gherardi-Donato, Luiz Jorge Pedrão

**Affiliations:** I Universidade de São Paulo. Ribeirão Preto, São Paulo, Brazil; II Universidade Federal de Mato Grosso. Cuiabá, Mato Grosso, Brazil

**Keywords:** Mindfulness, Meditation, Emotional Regulation, Education, Nursing Students, Atención Plena, Meditación, Regulación Emocional, Universidades, Estudiantes de Enfermería

## Abstract

**Objective:**

to identify the scientific evidence available in national and international literature on the relationships between nursing students’ mindfulness and emotional regulation.

**Methods:**

an integrative literature review, in four databases, with a time frame from January 2002 to December 2022. Articles in English, Spanish and Portuguese available in full were included.

**Results:**

the sample consisted of six original articles, predominantly with a quantitative approach, with cross-sectional designs standing out.

**Conclusion:**

the synthesis of scientific production revealed that there is a lack of research at national and international level on the subject with experimental and qualitative designs that enable conclusions about cause and effect and/or take into account subjective experiences of the applicability of mindfulness-based practice in nursing students’ emotional regulation.

## RESUMEN

**Objetivo:**

identificar la evidencia científica disponible en la literatura nacional e internacional sobre las relaciones entre mindfulness y regulación emocional en estudiantes de enfermería.

**Métodos:**

revisión integrativa de la literatura, en cuatro bases de datos, con un período de tiempo de enero de 2002 a diciembre de 2022. Se incluyeron artículos en inglés, español y português disponibles en su totalidad.

**Resultados:**

la muestra estuvo compuesta por seis artículos originales, predominantemente con enfoque cuantitativo, destacándose los diseños transversales.

**Conclusion:**

la síntesis de la producción científica reveló que faltan investigaciones a nivel nacional e internacional sobre el tema con diseños experimentales y cualitativos que permitan extraer conclusiones sobre causa y efecto y/o tomar en cuenta experiencias subjetivas de aplicabilidad de la práctica basada en mindfulness en la regulación emocional de estudiantes de enfermería.

## INTRODUCTION

Among the various undergraduate courses in health, nursing training has stood out in the scientific literature as related to an environment with high potential for emotional illness, due to the fact that healthcare constantly provides opportunities for experiences that generate excessive worry, fear and insecurity, as dealing with human beings and their suffering, constant observation of mentors in the practical field, the fear of making mistakes, in addition to the sources of stress linked to the professionalization process^(1, 2, 3)^.

Therefore, given the obstacles that nursing training encompasses, it is impossible, throughout this journey, to prevent students from going through challenging emotional experiences; therefore, in these moments, it is extremely important that they are able to skillfully manage the emotions activated^(1, 4)^.

Important scholars in the field^(5, 6, 7)^ understand that, to regulate emotions, it is necessary to use strategies that help the subject to deal with them as well as to control or reduce them correctly, increasing the chances of being faced appropriately, considering the social context of expression of emotion.

Among the currently existing therapeutic approaches for regulating emotions and managing stress, those based on mindfulness have been standing out scientifically among university students, in order to promote students’ mental health and well-being, which, consequently, will positively impact academic performance, considering that it encourages neuroplasticity and cognitive, socio-emotional and attentional development^(8, 9, 10, 11, 12)^.

The mindfulness construct was presented in the West at the end of the 20^th^ century by Jon Kabat-Zinn, with applications in the clinical area, which teaches that a way to better understand mindfulness is to idealize it as a mental process that involves awareness that arises when paying more attention to current events that are achieved without judgment and with acceptance, kindness towards oneself and others^(13)^.

Research^(14, 15, 16, 17, 18, 19, 20, 21)^ has demonstrated that mindfulness is closely related to more favorable physical and mental health habits, as individuals who have a higher level of mindfulness have more resources to regulate their emotions, which will help in coping with stress, anxiety, depression and fatigue, and can also improve resilience and mood. Therefore, the emotional regulation variable is of great importance in designing mindfulness.

In this way, it is expected that the results and analysis obtained from this research will collaborate in demonstrating the current scenario of scientific evidence on the relationships between nursing students’ mindfulness and emotional regulation. This could contribute information to improve studies in this area and encourage discussions about the importance of engaging training institutions and the entire teaching team in helping these students face adversities linked to professionalization, through strategies that enable the development of stress management skills, such as programs based on mindfulness.

## OBJECTIVE

To identify the scientific evidence available in national and international literature on the relationships between nursing students’ mindfulness and emotional regulation.

## METHOD

This is an integrative literature review, which makes it possible to synthesize the state of the art on a given topic through the identification and analysis of primary studies in a systematic way. Furthermore, this review method was chosen because it allows the simultaneous inclusion of studies with different research designs, which made it possible to increase the depth and scope of the general conclusions of the research problem of this investigation^(22)^.

To construct this integrative review, whose protocol was registered on the Figshare^(23)^ platform, six distinct stages were followed^(22)^: problem identification; literature search; categorization of selected studies; critical assessment; interpretation of results; and presentation of knowledge synthesis.

Based on the JBI review manual^(24)^, the research question was prepared applying the PICo strategy, represented by the acronym of the terms “P” for population/patient, “I” for phenomenon of interest and “Co” for context. In this study, P = nursing students, I = relationships between mindfulness and emotional regulation, Co = public and private universities. Therefore, the following research question was formulated: what is the evidence available in national and international literature on the relationships between nursing students’ mindfulness and emotional regulation?

The searches were carried out in June 2023 by two authors of this research. The national and international databases investigated, as they bring together and make available the main journals of interest for this research, were PubMed/MEDLINE from the National Library of Medicine, Cumulative Index to Nursing and Allied Health Literature (CINAHL), Scopus and Web of Science. Aiming to cover the entire theme, in addition to the controlled terms relevant to the dictionaries available in part of the databases (MeSH), CINAHL, DeCS and Emtree subjects, possible synonyms and Boolean operators for crossing were also included, considering the specificities of each database (Chart 1).

**Chart 1:** Database and Search strategies

Database	Search strategies
PubMed	(“*Atenção plena”* OR “*Consciência Plena”* OR “Mindfulness” OR “*Atención Plena”* OR “*Consciência”* OR “*Meditação”* OR “Meditation” OR “*Meditación*”) AND *(“Regulação Emocional”* OR “Emotional Regulation” OR “*Regulación Emocional”* OR “*Desregulação Emocional”* OR “Emotional Dysregulation” OR “*Desregulación Emocional”* OR “*Autorregulação Emocional”* OR “Emotional Self-Regulation” OR “*Autorregulación Emocional*” OR “*Regulação da Emoção”* OR “Emotion Regulation”) AND (“*Estudantes” OR “Alunos”* OR “Students” OR “*Estudiantes”* OR “*Alumno”* OR “*Estudantes Universitários”* OR “College Students” OR “*Estudantes de Graduação” OR “*Graduations Students” OR “*Estudiantes Graduado”* OR “*Graduando”* OR “Graduating” OR “*Graduarse”* OR “*Estudantes de Enfermagem”* OR “Students Nursing” OR “*Estudiantes de Enfermería”*)
CINAHL	(“Mindfulness” OR “Meditation”) AND (“Emotional Regulation” OR “Emotion Regulation” OR “Emotional Dysregulation” OR “Emotional Self-Regulation”) AND (“Students” OR “College Students” OR “Graduations Students” OR “Graduating” OR “Students Nursing”)
Scopus	“Mindfulness” OR “Meditation” AND “Emotional Regulation” OR “Emotion Regulation” OR “Emotional Dysregulation” OR “Emotional Self-Regulation” AND “Students” OR “College Students” OR “Graduations Students” OR “Graduating” OR “Students Nursing”
Web of Science	All=(((“Mindfulness” OR “Meditation”) AND (“Emotional Regulation” OR “Emotion Regulation” OR “Emotional Dysregulation” OR “Emotional Self-Regulation”) AND (“Students” OR “College Students” OR “Graduations Students” OR “Graduating” OR “Students Nursing”)))

Primary studies with abstracts published in Portuguese, English and Spanish, that address the relationships between nursing students’ mindfulness and emotional regulation, were eligible. Furthermore, to expand the screening of evidence, studies from the last 20 years were included – January 2002 to December 2022. Gray literature, experience reports, editorials, abstracts published in annals of scientific events, records unavailable in full, studies that do not respond to the research question and do not report data from nursing students separately from other students and new studies, after the end of the search period, were not included in the research.

## RESULTS

From reading titles and abstracts, 218 primary studies were pre-selected. Subsequently, duplicate records were identified, 110 of which were excluded at this stage. After removing duplications and based on inclusion criteria, 75 articles were chosen to be read in full. Subsequently, a total of 69 records were excluded because they were not found in full, did not investigate nursing students and did not specify the undergraduate course involved in the research. In the final sample, six studies were included.

**Figure 1:**
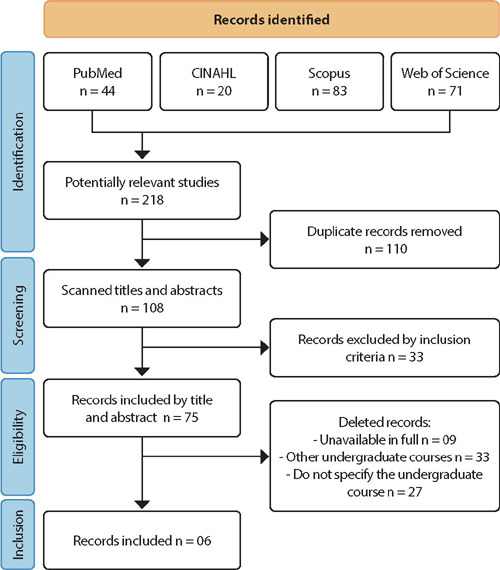
Flowchart of the study selection process adapted from the Preferred Reporting Items for Systematic Reviews and Meta-Analyses (PRISMA)^(25)^

Article reading and selection were carried out by two reviewers independently, and a third reviewer collaborated to resolve conflicts. The report of this review, containing all stages of the selection process, adapted for this research, was based on the Preferred Reporting Items for Systematic Reviews and Meta-Analyses (PRISMA)^(25)^ (Figure 1).

The information extracted from the selected studies, tabulated in a script developed for this purpose, covered author, year, country, objectives, design, number of participants and the main results and conclusions of each study (Chart 2).

**Chart 2:** Synthesis of primary studies included in the integrative review

Author, years, country	Objective	Design/number of participants	Main results and conclusion
Dubert *et al.,* 2016^(26)^ (USA)	Examine the relationship between mindfulness and emotional regulation in nursing students as well as the potential mediating role of working memory capacity in this relationship.	Cross-sectional n=85	There was a significant but relatively small positive correlation between dispositional mindfulness and emotional regulation (reappraisal). These results suggest that dispositional mindfulness has a direct effect on the use of reappraisal as an emotional regulation strategy and working memory capacity.
Chow *et al.,* 2020^(27)^ (China)	Develop a resilience building module and assess the effect of applying this module on the results of resilience, well-being and mindfulness in a sample of nursing students.	Sequential explanatory n=195	The second workshop of the module introduced approaches to stress management, with a particular focus on the use of mindfulness-based practices. Univariate and multivariate regression analyzes showed that dispositional mindfulness presented significant positive correlations with resilience. Only dispositional mindfulness remained significantly associated with resilience, indicating that this variable is a relevant independent predictor of resilience. Notably, the data above suggest that the level of dispositional mindfulness independently predicted resilience in this sample. Further research is recommended to explore the effects of a more intensive treatment program with a stronger focus on mindfulness and the inclusion of more activities in smaller groups.
Salvarani *et al.*, 2020^(28)^ (Italy)	Assess psychological distress in a sample of Italian nursing students and explore its relationship with sociodemographic and psychological factors, specifically dispositional mindfulness, difficulties in emotional regulation and empathy.	Cross-sectional n=622	Regarding the dimensions of dispositional mindfulness that predict the psychological performance of nursing students, participants with higher levels of describing (p = 0.016), acting with awareness (p < 0.001), non-judgment (p < 0.001) and not reactivity (p < 0.001) had lower levels of psychological distress. These results suggest that the inclusion of mindfulness-based interventions may be beneficial in protecting nursing students from stress.
Lee and Jang, 2021^(29)^ (South Korea)	Investigate the relationships between socio-cognitive mindfulness, emotional regulation, and positive and negative emotions of achievement among nursing students.	Cross-sectional n=459	Sociocognitive mindfulness correlated positively with reappraisal and negatively with suppression. Furthermore, reappraisal mediated the link between sociocognitive mindfulness and positive emotions, and suppression mediated the link between sociocognitive mindfulness and negative emotions. These findings suggest that sociocognitive mindfulness may be effective in regulating emotions among nursing students, improving reappraisal and reducing suppression.
Costa *et al.,* 2022^(30)^ (Brazil)	Investigate the correlation between dispositional mindfulness, emotional regulation and perceived stress and verify factors associated with dispositional mindfulness in nursing students.	Cross-sectional n=335	Moderate mean scores for dispositional mindfulness, emotional regulation and perceived stress were identified. There was no significant correlation between dispositional mindfulness, general emotional regulation score and perceived stress, with this first variable being weakly correlated with the emotional regulation dimension of emotion suppression. Thus, the results indicate a relationship between dispositional mindfulness only with the dimension of emotional regulation and suppression of emotions.
Noroozie and Mohebbi-Dehnavi, 2022^(31)^ (Iran)	Investigate and compare the effect of two educational methods based on mindfulness and cognitive emotional regulation strategies on the psychological well-being and anxiety of nursing and midwifery students.	Quasi-experimental Pre- and post-test n=30	The results of this study showed that mindfulness-based and cognitive emotional regulation training can reduce anxiety scores and increase psychological well-being scores in nursing and midwifery students before the comprehensive examination. Therefore, it is recommended to consider educational programs based on mindfulness practices and cognitive emotional regulation to promote the mental health and academic/professional success of this group of students.

For critical assessment of selected studies, we chose to assess the methodological quality proposed by McMaster University^(32)^, which consists of a tool that does not provide a scoring system for the general study assessment, only answers such as “yes”, “no” “not informed” and “not applicable”. In this review, the following components were assessed: was the objective clear?; was a review of relevant literature carried out on this topic?; describe the design; sample described in detail; justification for sample size presented; reliable outcome measures; valid outcome measures; intervention described in detail; contamination was avoided; simultaneous intervention was avoided; results reported in terms of statistical significance; adequate analysis methods; clinical importance was reported; report of participants who abandoned the study; conclusions consistent with the methods and results obtained. The extracted data were analyzed and presented in a descriptive and tabular form (Table 1).

**Table 1:** Analysis of the methodological quality of quantitative studies based on the tool proposed by McMaster University^(32)^. Brazil, 2023

	Dubert *et al.,* 2016^(26)^	Chow *et al.,* 2020^(27)^	Salvarani *et al.,* 2020^(28)^	Lee and Jang, 2021^(29)^	Costa *et al.,* 2022^(30)^	Noroozie and Mohebbi-Dehnavi, 2022^(31)^
Was the objective clear?	Y	Y	Y	Y	Y	Y
Was a review of relevant literature carried out on this topic?	Y	Y	Y	Y	Y	Y
Describe the design	CST	QES	CST	CST	CST	CST
Sample described in detail	Y	Y	Y	Y	Y	Y
Justification for sample size presented	Y	Y	Y	Y	Y	Y
Reliable outcome measures	Y	Y	Y	Y	Y	Y
Valid outcome measures	Y	Y	Y	Y	Y	Y
Intervention described in detail	NA	S	NA	NA	NA	Y
Contamination was avoided	NA	N	NA	NA	NA	N
Simultaneous intervention was avoided	NA	S	NA	NA	NA	Y
Results reported in terms of statistical significance	Y	Y	Y	Y	Y	Y
Adequate analysis methods	Y	Y	Y	Y	Y	Y
Clinical importance was reported	Y	Y	Y	Y	Y	Y
Report of participants who abandoned the study	NA	Y	NA	NA	NA	N
Conclusions consistent with the methods and results obtained	Y	Y	Y	Y	Y	Y

*Caption: Y - yes; N - no; NA - not applicable; CST - cross-sectional study; QES - quasi-experimental study.*

According to the synthesis of scientific knowledge assessed in this review (Chart 2), a total of six original articles were selected, with studies between 2016 and 2022, with higher frequencies in 2020 and 2022 (two studies/year).

The studies were produced in America, Asia and Europe, with emphasis on a greater number of productions in Asia (n=3). America covered two studies (n=2), one of which (n=1) was produced in Brazil. Regarding language, there was a predominance of articles in English (n=5). Studies took place in national and international nursing journals that address generic subjects (n=3), in multi-professional journals specializing in mindfulness (n=1) and in multi-professional journals with general themes in health (n=2). There was a predominance of a quantitative research approach, highlighting the use of cross-sectional methodology (n=4).

Based on the tool proposed by McMaster University^(32)^ (Table 1), all studies (n=06) presented: clarity in detailing the objectives; review of relevant literature on this topic; sample described in detail; justification for sample size; reliable or valid outcome measures; results reported in terms of statistical significance; adequate analysis methods; reported clinical importance and conclusions consistent with the methods; and results obtained. Of the studies included with an experimental design (n=2), both did not avoid sample contamination, and one (n=1) presented report of participants who abandoned the study.

## DISCUSSION

It was evidenced, in most of the articles included in this review, that mindfulness presented positive relationships in nursing students’ emotional regulation, being able to: provide a direct effect on the use of cognitive regulation of emotions as an emotional regulation strategy^(26)^ (n=1); independently predict resilience^(27)^ (n=1); predict lower levels of psychological distress^(28)^ (n=1); contribute to increasing the cognitive reappraisal of emotions and reducing the suppression of emotions^(29)^ (n=1); contribute to increasing the use of emotion suppression strategies^(30)^ (n=1); and cooperate in reducing anxiety scores and increasing psychological well-being scores^(31)^ (n=1).

In this study, it was highlighted that mindfulness has the potential to increase the use of cognitive reappraisal of emotions^(26, 29)^ as an emotional regulation strategy capable of mediating the link between nursing student mindfulness and positive emotions^(29)^.

These results are consistent with other studies that indicated positive effects of the relationship between mindfulness and emotional regulation strategies, as greater use of the strategy of cognitive reappraisal of emotions can favor fewer negative thoughts and less stress, with an impact on health and well-being^(33)^. Additionally, neuroscientific evidence suggests that the cognitive reappraisal strategy and mindfulness share the same neural circuits involved in emotional regulation^(34)^.

On the other hand, a cross-sectional study with nursing students, which appears in this review^(30)^, sought to investigate the correlation between dispositional mindfulness and emotional regulation through the Mindful Attention Awareness Scale and the Emotional Regulation Questionnaire. No relationship was identified between dispositional mindfulness and cognitive reappraisal of emotions, even though this variable is considered the operating mechanism of mindfulness interventions^(35)^. Only the suppression of emotions dimension was related, even if weakly, in a positive way with dispositional mindfulness^(30)^. A possible explanation for this finding may be linked to the lack of consensus in the literature regarding the concept and measurement instruments used in the assessment of such constructs^(33)^. Meta-analytic investigation revealed that research that correlated mindfulness and emotional regulation using the Mindful Attention Awareness Scale were those that presented the smallest magnitudes of effect in these relationships when compared to research that assessed mindfulness using other instruments^(36)^. These authors concluded that this could be due to the fact that the Mindful Attention Awareness Scale, in their assessment, does not emphasize emotional domains, as occurs with other scales, such as the Five Facet Mindfulness Questionnaire (FFMQ) and the Freiburg Mindfulness Inventory (FMI)^(36)^.

Corroborating this, research that analyzed the influence of emotional regulation strategies mindfulness, cognitive reappraisal and emotional suppression in the daily lives of university students revealed that the suppression of emotions can also be an effective emotional regulation strategy for some people in different situations, requiring a careful assessment of each case^(37)^.

Therefore, to better understand this mechanism, it is essential to conduct research that can provide robust evidence, such as experimental studies, which assess the operational characteristics of mindfulness and which allow analyzing the personal experience of practice, since quantitative research using self-report instruments predominates^(33)^.

Some investigations in this review reinforce the interactions of mindfulness and emotional regulation with other psychology concepts, such as resilience^(27)^, psychological distress^(28)^, stress^(30)^, anxiety and psychological well-being^(31)^.

This further strengthens the understanding that the relationships between both constructs are directly linked to psychic performance and mental health, as there is a growing body of evidence suggesting that intervention based on mindfulness acts positively on characteristics related to emotional regulation, as it contributes to reducing levels of stress and anxiety, improving psychological well-being and resilience in student populations and healthcare professionals^(10, 33, 38)^.

It is observed, among the studies presented here, that there was a predominance of the cross-sectional research design, in which prediction relationships, associations or correlations and mediation were recommended, which restricts conclusions about causality or that precedes in time^(26, 28, 29, 30)^. Furthermore, there is a lack of evidence with qualitative approaches to assess the subjectivity of experiences based on mindfulness among nursing students^(27)^, in addition to there being few quasi-experimental studies^(27, 31)^ and no experimental controlled clinical trials.

### Study limitations

Limitations include, even if searches were carried out in different databases, the possibility that some important journals in the area were left out. The decision not to include gray literature, such as dissertations and theses, may have contributed to the exclusion of important research. Another limitation of this review is the scarcity of scientific evidence available in national and international literature on the relationships between nursing students’ mindfulness and emotional regulation, and this review could be the starting point for this discussion, arousing the interest of scholars on the subject.

### Contributions to nursing, health, or public policy

The scenario of national and international literature on the relationships between nursing students’ mindfulness and emotional regulation was presented. As it is the first review study in this specialty with nursing students, it allowed a greater understanding of the topic.

The synthesis of scientific production identified areas of research that are still incipient. Among them, the low frequency of qualitative research to assess the subjectivity of experiences based on mindfulness among nursing students stands out. Furthermore, there are few experimental studies, making it impossible to obtain comprehensive conclusions regarding its applicability.

Therefore, given that exposure to various academic and clinical stressors is an inevitable aspect of nursing students’ experience, this review may guide the deepening and design of future investigations in this field of knowledge, in addition to encouraging discussions about the importance of training institutions and teaching teams investing in strategies, such as those based on mindfulness, to help these students cope with stressful events that trigger emotional dysregulation, which could have a direct positive influence on the academic and clinical performance of these students and indirectly on the performance of recipients of nursing care and healthcare services.

## CONCLUSION

In the scientific literature, it was almost entirely found that mindfulness showed positive relationships in nursing students’ emotional regulation. It was also observed that some studies in this review reinforce the interactions of mindfulness and emotional regulation with other psychological concepts, such as resilience, psychological distress, stress, anxiety and psychological well-being. However, scientific production was still incipient on the relationships of mindfulness in the emotional regulation of nursing students.

Therefore, it is strongly recommended that scholars interested in the subject use experimental and qualitative research designs that allow conclusions about cause and effect and/or take into account subjective experiences of the applicability of practices based on mindfulness in nursing students’ emotional regulation.
