# Supplementary material for: Nursing students’ mindfulness and emotional regulation: an integrative review
Source: Rev Bras Enferm. 2024 Sep 6;77(4):e20230466. doi: 10.1590/0034-7167-2023-0466 (PMC11382681; doi:10.1590/0034-7167-2023-0466)
Supplement: Translation

# Translation: Atenção plena e regulação emocional de graduandos de enfermagem: revisão integrativa

**DOI:** 10.1590/0034-7167-2023-0466pt

**Authors:** Daiana Alves Vendramel da Costa, Cássia Janne Nonato da Costa, Moisés Kogien, Larissa Bessani Hidalgo Gimenez, Edilaine Cristina da Silva Gherardi-Donato, Luiz Jorge Pedrão

**Affiliations:** I Universidade de São Paulo. Ribeirão Preto, São Paulo, Brasil; II Universidade Federal de Mato Grosso. Cuiabá, Mato Grosso, Brasil

**Keywords:** Atenção Plena, Meditação, Regulação Emocional, Educação Superior, Estudantes de Enfermagem

## Abstract

**Objetivo:**

identificar as evidências científicas disponíveis na literatura nacional e internacional sobre as relações entre atenção plena e regulação emocional de graduandos de enfermagem.

**Métodos:**

revisão integrativa de literatura, em quatro bases de dados, com recorte temporal de janeiro de 2002 a dezembro de 2022. Foram incluídos artigos em inglês, espanhol e português disponíveis na íntegra.

**Resultados:**

a amostra constituiu-se de seis artigos originais, predominantemente, de abordagem quantitativa, sobressaindo-se delineamentos de corte transversal.

**Conclusão:**

a síntese da produção científica revelou que há carência de pesquisas a nível nacional e internacional sobre a temática com delineamentos experimentais e qualitativos que possibilite conclusões sobre causa e efeito e/ou levem em consideração experiências subjetivas da aplicabilidade da prática baseada na atenção plena na regulação emocional de graduandos de enfermagem.

## INTRODUÇÃO

Entre os vários cursos de graduação na área da saúde, a formação em enfermagem vem se destacando na literatura científica como relacionada a um ambiente de alto potencial para adoecimento emocional, pelo fato de o cuidado em saúde oportunizar constantemente experiências que geram preocupação excessiva, medo e insegurança, como lidar com o ser humano e seu sofrimento, a observação constante do mentores no campo prático, o medo de errar, além das fontes de estresse ligadas ao processo de profissionalização^(1, 2, 3)^.

Dessa forma, diante dos obstáculos que abrange a formação em enfermagem, é impossível, ao longo dessa jornada, evitar que os estudantes passem por experiências emocionais desafiadoras; logo, nesses momentos, é de extrema importância que sejam capazes de gerenciar habilmente as emoções ativadas^(1, 4)^.

Importantes estudiosos da área^(5, 5, 7)^ compreendem que, para regular as emoções, é necessário lançar mão de estratégias que auxiliem o sujeito a lidar com elas, bem como dominá-las ou reduzi-las corretamente, elevando as chances de serem enfrentadas de modo apropriado, considerando o contexto social de expressão da emoção.

Entre as abordagens terapêuticas existentes, na atualidade, para regulação das emoções e manejo do estresse, as baseadas na atenção plena vêm se destacando cientificamente entre universitários, a fim de promover a saúde mental e bem-estar dos estudantes, o que, consequentemente, impactará positivamente o desempenho acadêmico, tendo em vista que estimula a neuroplasticidade e o desenvolvimento cognitivo, socioemocional e atencional^(8, 9, 10, 11, 12)^.

O construto atenção plena foi apresentado no ocidente no final do século XX por Jon Kabat-Zinn, com aplicações na área clínica, o qual ensina que um jeito para melhor compreender a atenção plena é idealizá-la como processo mental que envolve a consciência que nasce ao prestar atenção com mais propriedade aos acontecimentos do presente que são alcançadas sem julgamento e com aceitação, gentileza consigo e com os outros^(13)^.

Pesquisas^(14, 15, 16, 17, 18, 19, 20, 21)^ vêm demonstrando que a atenção plena está intimamente relacionada a hábitos mais favoráveis de saúde física e mental, pois o sujeito que possui nível mais elevado de atenção plena dispõe de mais recursos para regular as emoções, o que auxiliará no enfrentamento do estresse, ansiedade, depressão e fadiga, podendo, também, aprimorar a resiliência e o humor. Por isso, a variável regulação emocional tem grande importância no delineamento da atenção plena.

Desse modo, espera-se que os resultados e a análise obtida a partir desta pesquisa colaborem ao demonstrar o atual cenário de evidências científicas sobre as relações entre atenção plena e regulação emocional de graduandos de enfermagem. Isso poderá contribuir com informações para o aprimoramento dos estudos nessa área e fomentar discussões sobre a importância do engajamento das instituições formadoras e de toda equipe de ensino no auxílio desses alunos no enfrentamento das adversidades atreladas à profissionalização, através de estratégias que possibilitem o desenvolvimento de habilidades para o gerenciamento do estresse, tais como os programas baseados na atenção plena.

## OBJETIVO

Identificar as evidências científicas disponíveis na literatura nacional e internacional sobre as relações entre atenção plena e regulação emocional de graduandos de enfermagem.

## MÉTODO

Trata-se de revisão integrativa da literatura, a qual possibilita sintetizar o estado da arte sobre determinada temática através da identificação e análise de estudos primários de modo sistematizado. Ademais, esse método de revisão foi escolhido por permitir a inclusão simultânea de estudos com diversos delineamentos de pesquisas, o que possibilitou aumentar a profundidade e a abrangência das conclusões gerais do problema de pesquisa desta investigação^(22)^.

Para construção desta revisão integrativa, cujo protocolo foi registrado na plataforma Figshare^(23)^, percorreram-se seis etapas distintas^(22)^: identificação do problema; busca na literatura; categorização dos estudos selecionados; avaliação crítica; interpretação dos resultados; e apresentação da síntese do conhecimento.

Fundamentada no manual de revisões do JBI^(24)^, a questão de pesquisa foi elaborada aplicando-se a estratégia PICo, representada pelo acrônimo dos termos em inglês “P” para população/patient/paciente, “I” para *phenomenon of interest/fenômeno* de interesse e “Co” para context/contexto. Neste estudo, P = estudantes do curso de graduação em enfermagem, I = relações da atenção plena na regulação emocional, Co = universidades públicas e privadas. Desse modo, formulou-se a seguinte questão de pesquisa: quais são as evidências disponíveis na literatura nacional e internacional sobre as relações entre atenção plena e regulação emocional de graduandos de enfermagem?

As buscas foram realizadas no mês de junho de 2023 por dois autores da presente pesquisa. As bases de dados nacionais e internacionais investigadas, por reunirem e disponibilizarem os principais periódicos de interesse para esta pesquisa, foram PubMed/MEDLINE da *National Library of Medicine, Cumulative Index to Nursing and Allied Health Literature* (CINAHL), Scopus e *Web of Science.* Objetivando abarcar toda a temática, além dos termos controlados pertinentes aos dicionários disponíveis em parte das bases de dados (MeSH), assuntos CINAHL, DeCS e Emtree, também foram incluídos os possíveis sinônimos e operadores booleanos para o cruzamento, considerando as especificidades de cada base de dados (Quadro 1).

**Quadro 1:** Bases de dados e estratégias de busca

Bases de dados	Estratégias de busca
PubMed	(“Atenção plena” *OR “*Consciência Plena” *OR “Mindfulness” OR “Atención Plena” OR “*Consciência” *OR “*Meditação” *OR “Meditation” OR “Meditación”*) *AND* (“Regulação Emocional” *OR “Emotional Regulation” OR “Regulación Emocional” OR* “Desregulação Emocional” *OR “EmotionalDysregulation” OR “Desregulación Emocional” OR “Autorr*egulação Emocio*nal” OR “EmotionalSelf-Regulation” OR “Autorregulación Emocional” OR “*Regulação da Emoção” *OR “Emotion Regulation”*) *AND* (“Estudantes” *OR “*Alunos” *OR “Students” OR “Estudiantes” OR “Alumno” OR “*Estudantes Universitários” *OR “College Students” OR “*Estudantes de Graduação” *OR “Graduations Students” OR “Estudiantes Graduado” OR “*Graduando” *OR “Graduating” OR “Graduarse” OR “*Estudantes de Enfermagem” *OR “Students Nursing” OR “Estudiantes de Enfermería*”)
CINAHL	(“*Mindfulness” OR “Meditation*”) *AND* (“*Emotional Regulation” OR “Emotion Regulation” OR “Emotional Dysregulation” OR “Emotional Self-Regulation*”) *AND* (“*Students” OR “College Students” OR “Graduations Students” OR “Graduating” OR “Students Nursing*”)
Scopus	*“Mindfulness” OR “Meditation “AND “Emotional Regulation” OR “Emotion Regulation” OR “Emotional Dysregulation” OR “Emotional Self-Regulation “AND “Students” OR “College Students” OR “Graduations Students” OR “Graduating” OR “Students Nursing*”
*Web of Science*	*All*=(((“*Mindfulness” OR “Meditation*”) *AND* (“*Emotional Regulation” OR “Emotion Regulation” OR “Emotional Dysregulation” OR “Emotional Self-Regulation*”) *AND* (“*Students” OR “CollegeStudents” OR “GraduationsStudents” OR “Graduating” OR “Students Nursing*”)))

Foram elegíveis estudos primários com resumos publicados nos idiomas português, inglês e espanhol, artigos que abordem sobre as relações entre atenção plena e regulação emocional de graduandos de enfermagem. Além disso, para ampliar o rastreio das evidências, foram incluídas publicações dos últimos 20 anos – janeiro de 2002 a dezembro de 2022. Literatura cinzenta, relatos de experiência, editoriais, resumos publicados em anais de eventos científicos, registros indisponíveis na íntegra, estudos que não respondam ao questionamento de pesquisa e que não relatem dados de graduandos de enfermagem separadamente de outros estudantes e novos estudos, após terminado o período de busca, não foram incluídos na pesquisa.

## RESULTADOS

A partir da leitura dos títulos e resumos, pré-selecionaram-se 218 estudos primários. Na sequência, foram identificados os registros duplicados, sendo 110 excluídos nessa etapa. Após remoção das duplicações e por critérios de inclusão, foram eleitos 75 artigos para leitura na íntegra. Posteriormente, um total de 69 registros foram excluídos por não terem sido encontrados na íntegra, não investigarem graduandos de enfermagem e não especificarem o curso de graduação envolvido na pesquisa. Na amostra final, incluíram-se seis estudos.

A leitura e a seleção dos artigos foram realizadas por dois revisores de modo independente, e um terceiro revisor colaborou para a solução de conflitos. O relato desta revisão, contendo todas as etapas do processo de seleção, adaptado para esta pesquisa, baseou-se no *Preferred Reporting Items for Systematic Reviews and Meta-Analyses* (PRISMA)^(25)^ (Figura 1).

**Figura 1:** Fluxograma do processo de seleção dos estudos adaptado *do Preferred Reporting Items for Systematic Reviews and Meta-Analyses* (PRISMA)^(25)^

As informações extraídas dos estudos selecionados, tabuladas em roteiro desenvolvido para este fim, abrangeram autor, ano, país, os objetivos, delineamento, número de participantes e os principais resultados e conclusões de cada estudo (Quadro 2).

**Quadro 2:** Síntese dos estudos primários incluídos na revisão integrativa

Autor, ano, país	Objetivo	Delineamento/número de participantes	Principais resultados e conclusão
Dubert *et al.,* 2016^(26)^ (EUA)	Examinar a relação entre atenção plena e regulação emocional em estudantes de enfermagem, bem como o potencial papel mediador da capacidade de memória de trabalho nessa relação.	Transversal n=85	Houve correlação positiva significativa, mas relativamente pequena, entre atenção plena disposicional e regulação emocional (reavaliação). Tais resultados sugerem que atenção plena disposicional tem um efeito direto sobre o uso da reavaliação como estratégia de regulação emocional e capacidade de memória de trabalho.
Chow *et al.,* 2020^(27)^ (China)	Desenvolver um módulo de construção de resiliência e avaliar o efeito da aplicação desse módulo sobre os resultados de resiliência, bem-estar e atenção plena em amostra de estudantes universitários de enfermagem.	Sequencial explanatório n=195	A segunda oficina do módulo introduziu abordagens para gerenciamento do estresse, com foco particular no uso de práticas baseadas na atenção plena. Análises de regressão univariada e multivariada evidenciaram que a atenção plena disposicional apresentou correlações positivas significativas com a resiliência. Apenas a atenção plena disposicional permaneceu significativamente associada com resiliência, indicando que essa variável é um preditor independente relevante de resiliência. Notavelmente, os dados expostos sugerem que o nível de atenção plena disposicional previu independentemente a resiliência nesta amostra. Mais pesquisas são recomendadas para explorar os efeitos de programa de tratamento mais intensivo com foco mais forte na atenção plena e a inclusão de mais atividades em grupos menores.
Salvarani *et al.*, 2020^(28)^ (Itália)	Avaliar o sofrimento psicológico em amostra de estudantes de enfermagem italianos e explorar sua relação com fatores sociodemográficos e psicológicos, especificamente atenção plena disposicional, dificuldades de regulação emocional e empatia.	Transversal n=622	Em relação às dimensões de atenção plena disposicional que predizem o desempenho psicológico dos estudantes de enfermagem, os participantes com níveis mais altos de descrever (p = 0,016), agir com consciência (p < 0,001), não julgamento (p < 0,001) e não reatividade (p < 0,001) tiveram níveis mais baixos de sofrimento psicológico. Esses resultados sugerem que a inclusão de intervenções baseadas na atenção plena pode ser benéfica na proteção dos estudantes de enfermagem ao estresse.
Lee e Jang, 2021^(29)^ (Coréia do Sul)	Investigar as relações entre atenção plena sociocognitiva, regulação emocional e emoções positivas e negativas de realização entre estudantes universitários de enfermagem.	Transversal n=459	Atenção plena sociocognitiva correlacionou-se positivamente com a reavaliação e negativamente com a supressão. Além disso, a reavaliação mediou a ligação entre atenção plena sociocognitiva e as emoções positivas, e a supressão mediou a ligação entre atenção plena sociocognitiva e as emoções negativas. Esses achados sugerem que a atenção plena sociocognitiva pode ser eficaz na regulação das emoções entre estudantes de enfermagem, melhorando a reavaliação e reduzindo a supressão.
Costa *et al.,* 2022^(30)^ (Brasil)	Investigar a correlação entre atenção plena disposicional, regulação emocional e estresse percebido e verificar fatores associados à atenção plena disposicional em estudantes de enfermagem.	Transversal n=335	Identificaram-se escores médios moderados de atenção plena disposicional, regulação emocional e estresse percebido. Não foi evidenciada correlação significativa entre atenção plena disposicional, o escore geral de regulação emocional e estresse percebido, tendo essa primeira variável se correlacionado fracamente com a dimensão de regulação emocional supressão das emoções. Assim, os resultados sinalizam relação entre a atenção plena disposicional apenas com a dimensão de regulação emocional e supressão das emoções.
Noroozie e Mohebbi-Dehnavi, 2022^(31)^ (Irã)	Investigar e comparar o efeito de dois métodos educacionais baseados na atenção plena e estratégias de regulação emocional cognitiva no bem-estar psicológico e na ansiedade de estudantes de enfermagem e obstetrícia.	Quase-experimental Pré- e pós-teste n=30	Os resultados do presente estudo mostraram que o treinamento baseado na atenção plena e de regulação emocional cognitiva pode reduzir os escores de ansiedade e aumentar os escores de bem-estar psicológico em estudantes de enfermagem e obstetrícia antes do exame abrangente. Para tanto, recomenda-se considerar programas educativos baseados em práticas de atenção plena e regulação emocional cognitiva para promover a saúde mental e o sucesso acadêmico/profissional desse grupo de alunos.

Para avaliação crítica dos estudos selecionados, optou-se pela avaliação da qualidade metodológica proposta pela Universidade de McMaster^(32)^, que consiste em ferramenta que não disponibiliza um sistema de pontuação para avaliação geral do estudo, apenas respostas do tipo “sim”, “não”, “não informado” e “não se aplica” Nesta revisão, foram avaliados os seguintes componentes: objetivo estava claro?; realizada revisão da literatura relevante neste tema?; descreva o desenho; amostra descrita detalhadamente; apresentada justificativa para tamanho amostral; medidas de desfechos confiáveis; medidas de desfechos válidas; intervenção descrita de forma detalhada; contaminação foi evitada; intervenção simultânea foi evitada; resultados relatados em termos de significância estatística; métodos de análise adequados; importância clínica foi relatada; relato de participantes que abandonaram o estudo; conclusões coerentes com os métodos e resultados obtidos. Os dados extraídos foram analisados e apresentados de forma descritiva e tabular (Tabela 1).

**Tabela 1:** Análise da qualidade metodológica dos estudos quantitativos fundamentada na ferramenta proposta pela Universidade de McMaster^(32)^. Brasil, 2023

	Dubert *et al.,* 2016^(26)^	Chow *et al.,* 2020^(27)^	Salvarani *et al.,* 2020^(28)^	Lee e Jang, 2021^(29)^	Costa *et al.,* 2022^(30)^	Noroozie e Mohebbi-Dehnavi, 2022^(31)^
Objetivo estava claro?	S	S	S	S	S	S
Realizada revisão da literatura relevante neste tema?	S	S	S	S	S	S
Descreva o desenho	ET	EQE	ET	ET	ET	EQE
Amostra descrita detalhadamente	S	S	S	S	S	S
Apresentada justificativa para tamanho amostral	S	S	S	S	S	S
Medidas de desfechos confiáveis	S	S	S	S	S	S
Medidas de desfechos válidas	S	S	S	S	S	S
Intervenção descrita de forma detalhada	NA	S	NA	NA	NA	S
Contaminação foi evitada	NA	N	NA	NA	NA	N
Intervenção simultânea foi evitada	NA	S	NA	NA	NA	S
Resultados relatados em termos de significância estatística	S	S	S	S	S	S
Métodos de análise adequados	S	S	S	S	S	S
Importância clínica foi relatada	S	S	S	S	S	S
Relato de participantes que abandonaram o estudo	NA	S	NA	NA	NA	N
Conclusões coerentes com os métodos e resultados obtidos	S	S	S	S	S	S

*Legenda: S - sim; N - não; NA - não se aplica; ET - estudo transversal; EQE - estudo quase-experimental.*

Conforme a síntese de conhecimento científico avaliada nesta revisão (Quadro 2), o total de seis artigos originais foram selecionados, com publicações entre 2016 e 2022, com frequências maiores nos anos de 2020 e 2022 (duas produções/ano).

Os estudos foram produzidos nos continentes americano, asiático e europeu, com destaque para maior número de produções na Asia (n=3). O continente americano abrangeu dois estudos (n=2), sendo um (n=1) produzido no Brasil. Quanto ao idioma, notou-se predomínio de artigos na língua inglesa (n=5). As publicações ocorreram em revistas de enfermagem nacionais e internacionais que abordam assuntos genéricos (n=3), em revistas multiprofissionais especializadas em atenção plena (n=1) e em revistas multiprofissionais com temas gerais na área da saúde (n=2). Houve predomínio de abordagem quantitativa de pesquisa, sobressaindo-se uso de metodologia de corte transversal (n=4).

Com base na ferramenta proposta pela Universidade de McMaster^(32)^ (Tabela 1), todos os estudos (n=06) apresentaram: clareza no detalhamento dos objetivos; revisão da literatura relevante neste tema; amostra descrita detalhadamente; justificativa para tamanho amostrai; medidas de desfechos confiáveis ou válidas; resultados relatados em termos de significância estatística; métodos de análise adequados; importância clínica relatada e conclusões coerentes com os métodos; e resultados obtidos. Dos estudos incluídos com delineamento experimental (n=2), ambos não evitaram contaminação da amostra, e um (n=1) apresentou relato de participantes que abandonaram o estudo.

## DISCUSSÃO

Foi evidenciado, na maioria dos artigos inclusos nesta revisão, que a atenção plena apresentou relações positivas na regulação emocional de graduandos de enfermagem, sendo capaz de: proporcionar efeito direto sobre o uso da regulação cognitiva das emoções como estratégia de regulação emocional^(26)^ (n=1); predizer de modo independente a resiliência^(27)^ (n=1); predizer níveis mais baixos de sofrimento psicológico^(28)^ (n=1); contribuir no aumento da reavaliação cognitiva das emoções e redução da supressão das emoções^(29)^ (n=1); contribuir no aumento do uso das estratégias de supressão das emoções^(30)^ (n=1); e cooperar na redução de escores de ansiedade e aumentar os escores de bem-estar psicológico^(31)^ (n=1).

Neste estudo, destacou-se que a atenção plena tem potencial para aumentar o uso da reavaliação cognitiva das emoções^(26, 29)^ como estratégia de regulação emocional capaz de mediar a ligação entre atenção plena e emoções positivas de estudantes de enfermagem^(29)^.

Esses resultados são consistentes com outros estudos que indicaram efeitos positivos da relação entre atenção plena e estratégias de regulação emocional, como o maior uso da estratégia de reavaliação cognitiva das emoções pode favorecer menos pensamentos negativos, menor estresse, com impacto na saúde e no bem-estar^(33)^. Adicionalmente, provas neurocientíficas sugerem que a estratégia de reavaliação cognitiva e a atenção plena compartilham os mesmos circuitos neurais envolvidos na regulação emocional^(34)^.

Por outro lado, estudo de delineamento transversal com acadêmicos de enfermagem, que consta nesta revisão^(30)^, buscou investigar a correlação entre atenção plena disposicional e regulação emocional por meio da *Mindful Attention Awareness Scale* e do *Emotional Regulation Questionnaire.* Não identificou-se relação entre atenção plena disposicional e reavaliação cognitiva das emoções, mesmo essa variável sendo considerada o mecanismo de operação das intervenções de atenção plena^(35)^. Somente a dimensão supressão das emoções relacionou-se, mesmo que fracamente, de maneira positiva com atenção plena disposicional^(30)^. Possível explicação para esse achado talvez esteja atrelada à não existência de um consenso na literatura referente ao conceito e instrumentos de aferição empregados na avaliação de tais construtos^(33)^. Investigação metanalítica revelou que pesquisas que correlacionaram atenção plena e regulação emocional utilizando a *Mindful Attention Awareness Scale* foram aquelas que apresentaram as menores magnitudes de efeito nessas relações, quando comparadas com pesquisas que avaliaram atenção plena por meio de outros instrumentos^(36)^. Esses autores concluíram que isso poderia se dar pelo fato de a *Mindful Attention Awareness Scale,* em sua avaliação, não enfatizar domínios emocionais, como ocorre, por exemplo, com outras escalas, como a *Five Facet Mindfulness Questionnaire* (FFMQ) e a *Freiburg Mindfulness Inventory* (FMI)^(36)^.

Corroborando isso, pesquisa que analisou a influência das estratégias de regulação emocional atenção plena, reavaliação cognitiva e supressão emocional na vida diária de estudantes universitários revelou que a supressão das emoções pode mostrar-se também como estratégia de regulação emocional efetiva para algumas pessoas em diferentes situações, demandando uma avaliação criteriosa de cada caso^(37)^.

Portanto, para melhor entendimento desse mecanismo, é imprescindível a condução de pesquisas que possam trazer evidências robustas, como estudos de cunho experimental, que avaliem as características operacionais da atenção plena e que permitam analisar a experiência pessoal da prática, uma vez que predominam pesquisas de abordagem quantitativa com uso de instrumentos de autorrelato^(33)^.

Algumas investigações desta revisão reforçam as interações da atenção plena e regulação emocional com outros conceitos da psicologia, como resiliência^(27)^, sofrimento psicológico^(28)^, estresse^(30)^, ansiedade e bem-estar psicológico^(31)^.

Isso robustece ainda mais o entendimento de que as relações entre ambos os construtos estão diretamente ligadas ao desempenho psíquico e à saúde mental, pois há um crescente corpo de evidências sugerindo que intervenção baseada na atenção plena age positivamente nas características relacionadas à regulação emocional, uma vez que contribui para reduzir níveis de estresse e ansiedade, melhorar o bem-estar psicológico e resiliência nas populações de estudantes e profissionais da área da saúde^(10, 33, 38)^.

Observa-se, entre os estudos aqui apresentados, que houve predomínio do desenho transversal de pesquisa, nos quais foram recomendadas relações de predição, associações ou correlações e mediação, o que restringe conclusões sobre causalidade ou que anteceda no tempo^(26, 28, 29, 30)^. Além disso, há escassez de evidências com abordagens qualitativas para avaliar a subjetividade das experiências baseadas na atenção plena entre graduandos de enfermagem^(27)^, além de haver poucos estudos de delineamento quase-experimental^(27, 31)^ e nenhum experimental do tipo ensaio clínico controlado.

### Limitações do estudo

As limitações incluem, mesmo que tenham sido realizadas buscas em distintas bases de dados, a possibilidade de alguns periódicos importantes na área terem ficado de fora. A decisão de não incluir literatura cinzenta, como dissertações e teses, pode ter contribuído para a exclusão de pesquisas importantes. Outra limitação da presente revisão consiste na escassez de evidências científicas disponíveis na literatura nacional e internacional sobre as relações entre atenção plena e regulação emocional de graduandos de enfermagem, podendo esta revisão ser o marco inicial dessa discussão, despertando o interesse dos estudiosos no assunto.

### Contribuições para a área da enfermagem, saúde ou política pública

Foi apresentado o cenário da literatura nacional e internacional sobre as relações entre atenção plena e regulação emocional de graduandos de enfermagem. Por ser o primeiro estudo de revisão nesta especialidade com estudantes de enfermagem, permitiu um maior entendimento sobre a temática.

A síntese da produção científica identificou áreas de investigação ainda incipientes. Entre elas, destaca-se a baixa frequência de pesquisas qualitativas para avaliar a subjetividade das experiências baseadas na atenção plena entre graduandos de enfermagem. Além disso, há poucos estudos de cunho experimental, não sendo possível obter conclusões abrangentes a respeito de sua aplicabilidade.

Portanto, tendo em vista que a exposição a vários estressores acadêmico e clínico seja um aspecto inevitável da experiência dos graduandos de enfermagem, esta revisão poderá nortear o aprofundamento e delineamento de futuras investigações nesse campo do conhecimento, além de fomentar discussões sobre a importância de as instituições formadoras e equipe de ensino investirem em estratégias, tais como as baseadas em atenção plena, no auxílio desses alunos para enfrentamento dos eventos estressantes disparadores de desregulação emocional, o que poderá exercer influência positiva direta no desempenho acadêmico e clínico desses estudantes e indiretamente na performance junto a destinatários do cuidado de enfermagem e os serviços de saúde.

## CONCLUSÃO

Foi encontrado, na literatura científica, quase na totalidade que a atenção plena apresentou relações positivas na regulação emocional de graduandos de enfermagem. Observou-se, também, que alguns estudos desta revisão reforçam as interações da atenção plena e regulação emocional com outros conceitos da psicologia, como resiliência, sofrimento psicológico, estresse, ansiedade e bem-estar psicológico. No entanto, a produção científica mostrou-se ainda incipiente sobre as relações de atenção plena na regulação emocional de acadêmicos de enfermagem.

Assim, recomenda-se fortemente que estudiosos interessados no assunto utilizem delineamentos de pesquisas experimentais e qualitativos que permitam conclusões sobre causa e efeito e/ou levem em consideração experiências subjetivas da aplicabilidade de práticas baseadas na atenção plena na regulação emocional de graduandos de enfermagem.
